# Burning Mouth Syndrome in Association With Angular Cheilitis: A Case Report

**DOI:** 10.7759/cureus.67407

**Published:** 2024-08-21

**Authors:** Palak S Bhaiyya, Prasanna R Sonar, Priyanka Paul, Swamini S Gabhane, Anushree Rathi, Pooja D Dhole

**Affiliations:** 1 Dentistry, Sharad Pawar Dental College and Hospital, Datta Meghe Institute of Higher Education and Research (Deemed to be University), Wardha, IND; 2 Oral Medicine and Radiology, Sharad Pawar Dental College and Hospital, Datta Meghe Institute of Higher Education and Research (Deemed to be University), Wardha, IND; 3 Public Health Dentistry, Sharad Pawar Dental College and Hospital, Datta Meghe Institute of Higher Education and Research (Deemed to be University), Wardha, IND; 4 Orthodontics and Dentofacial Orthopaedics, Seema Dental College and Hospital, Rishikesh, IND; 5 Oral Medicine and Radiology, Vidharbha Youth Welfare Society Dental College, Amravati, IND

**Keywords:** ayurvedic medicine, ozonated oil, triphala, angular cheilitis, burning mouth syndrome

## Abstract

A burning sensation in the mouth without any obvious mucosal alterations is the hallmark of burning mouth syndrome, a chronic pain syndrome. BMS can worsen pain if it coexists with angular cheilitis, a condition characterized by inflammation around the corners of the mouth. Conventional therapies for angular cheilitis and burning mouth syndrome sometimes have unfavorable side effects and offer only little relief. When ozone is combined with oil, it accelerates tissue repair and oxygenation while destroying germs, fungi, and viruses. Triphala is a traditional treatment for oral health problems because of its anti-inflammatory, antioxidant, and antibacterial qualities. The effectiveness of alternative medicines, particularly ozonated oil, and triphala, a traditional herbal combination, in treating these diseases is examined in this case study. A 72-year-old woman reported a burning sensation in her mouth. The patient described the prolonged heat or burning sensation in the anterior two-thirds of her tongue. She was diagnosed with angular cheilitis and burning mouth syndrome. Conventional treatments, such as topical steroids and antifungal drugs, have not been effective. The patient was instructed to apply ozonated oil topically to the affected regions twice daily and to rinse their mouth with triphala. The patient reported full healing of the angular cheilitis lesions and considerable alleviation from burning feelings following two weeks of therapy. The patient noticed a significant decrease in the burning sensation in her mouth, characterized by a lack of discomfort, irritation, or pain. Throughout the treatment, no side effects were seen. According to this case study, ozonated oil and triphala may be useful in treating the symptoms of angular cheilitis and burning mouth syndrome, providing an alternative to traditional treatments.

## Introduction

Burning pain in the tongue or oral mucous membranes, typically absent from concomitant clinical and lab manifestations, is known as burning mouth syndrome (BMS) [1]. People suffering from this condition frequently report several oral problems, such as burning, dryness, and alteration in taste [2]. Women experience burning mouth problems more frequently than males, particularly after menopause [3]. Most of the time, patients wake up feeling fine but over their entire day towards the evening, their complaints get worse. Over 50% of individuals with BMS have discomfort that starts on its own without any clear trigger [4]. Usually affecting more than one oral location, the burning sensation is generally seen over the anterior two-thirds of the tongue, anterior hard palate, and lower lip [3]. Frequently, facial skin is unaffected. Patients with oral pain that is burning in type, often experience mood swings, such as impatience, anxiety, and melancholy, possibly due to sleep disruptions, ongoing discomfort, or both. Similar to other neuropathic pain syndromes, burning mouth syndrome is often treated medically by addressing its symptoms.

A distinctive diagnosis for a skin inflammation with a variety of etiologies that manifests at the labial commissure, or the angle of the mouth, is angular cheilitis (AC). The term "angular," also known as commissural, describes a confined inflammation of the lips [4]. Particular stressors can particularly affect the commissures. Cheilitis can result from exposure to chemicals, the environment, or pathogenic agents; they can also indicate an underlying illness, inadequacy, or disorder. It is important to comprehend these structural alterations to successfully treat and manage the resulting problems with oral health [5].

BMS and AC are distinct yet often overlapping oral conditions that can significantly impact a patient's quality of life. BMS is characterized by a persistent burning sensation in the oral mucosa, often without visible clinical signs. Its etiology is multifactorial, involving neuropathic, psychological, and hormonal factors. AC, on the other hand, presents as inflammation and cracking at the corners of the mouth, often caused by fungal or bacterial infections, nutritional deficiencies, or local irritants [6].

The co-occurrence of these two conditions can complicate diagnosis and management. While BMS is primarily a neuropathic pain disorder, AC typically involves a more straightforward infectious or inflammatory process. However, the symptoms of both conditions can exacerbate each other, leading to a challenging clinical scenario. This case report aims to explore a patient presenting with both BMS and AC, highlighting the diagnostic challenges, treatment strategies, and potential underlying connections between these conditions. This case highlights the likely effectiveness of integrative therapies like triphala and ozonated oil in managing complex oral conditions such as BMS and AC.

## Case presentation

**Figure 1:**
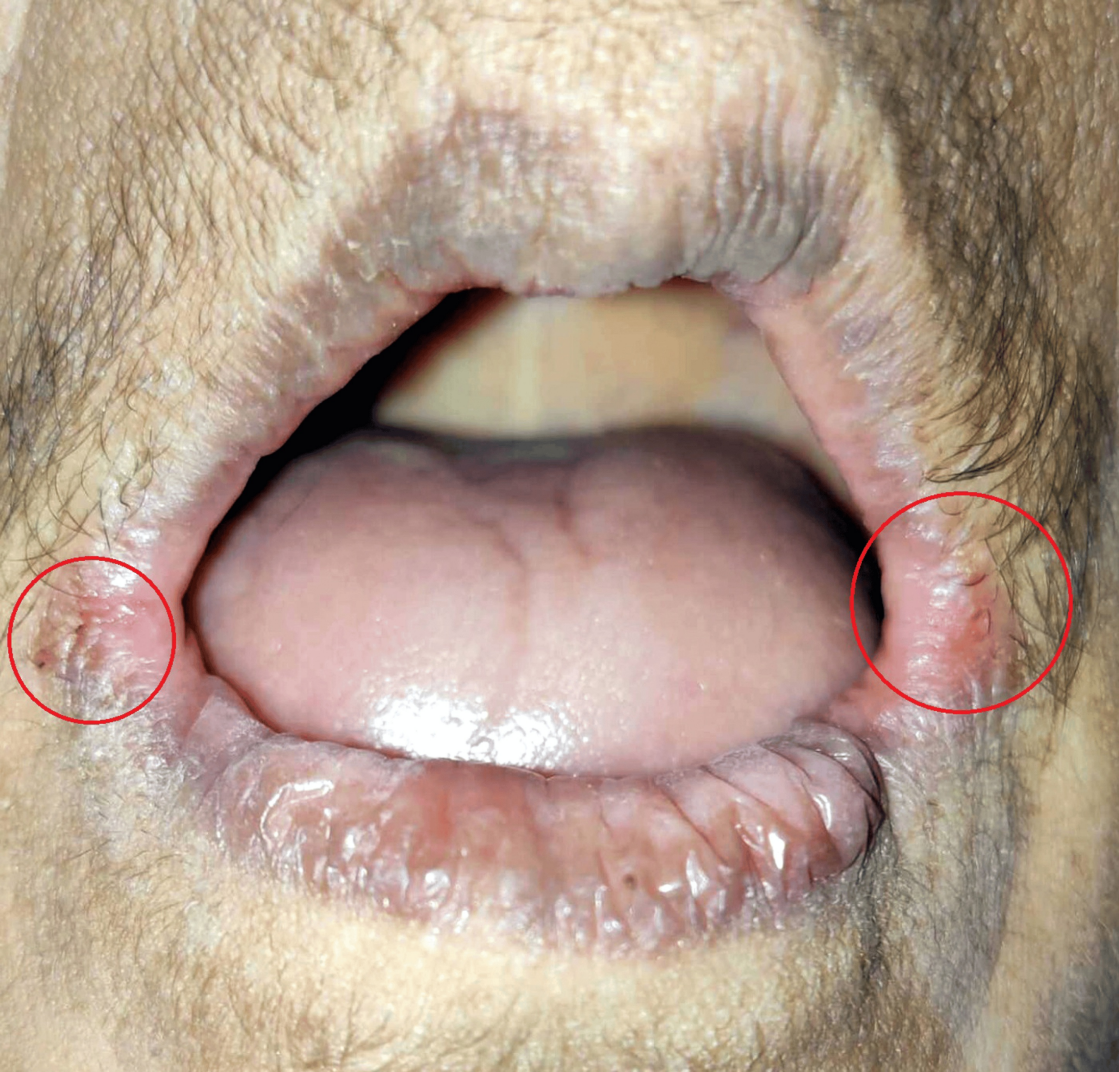
Extraoral photo showing angular cheilitis. Image credits: Prasanna Sonar

A 72-year-old female patient arrived at the dental hospital complaining primarily of missing teeth and a burning sensation throughout her buccal mucosa and tongue over the past two years. The patient said that she was experiencing burning throughout the anterior two-thirds of her tongue. She complained an increase in the burning sensation while eating spicy food, which was relieved on drinking water. The patient did not give any history of dryness and alteration in taste. There was no significant systemic history, nor was there any habit history. She said that she did not take any medicine. On examination, the patient seemed healthy, cooperative, conscious, and well-oriented to time, place, and person. Extraoral examination revealed erythema, or redness, in the corners of the mouth. The lesion was suggestive of the angular cheilitis. Figure 1 illustrates the clinical presentation of the lesion present on the corner of the lips bilaterally. On intraoral examination, the maxillary and mandibular arches were fully edentulous, and the oral mucosa was found to be normal and healthy with no mucosal abnormalities. 

**Figure 2:**
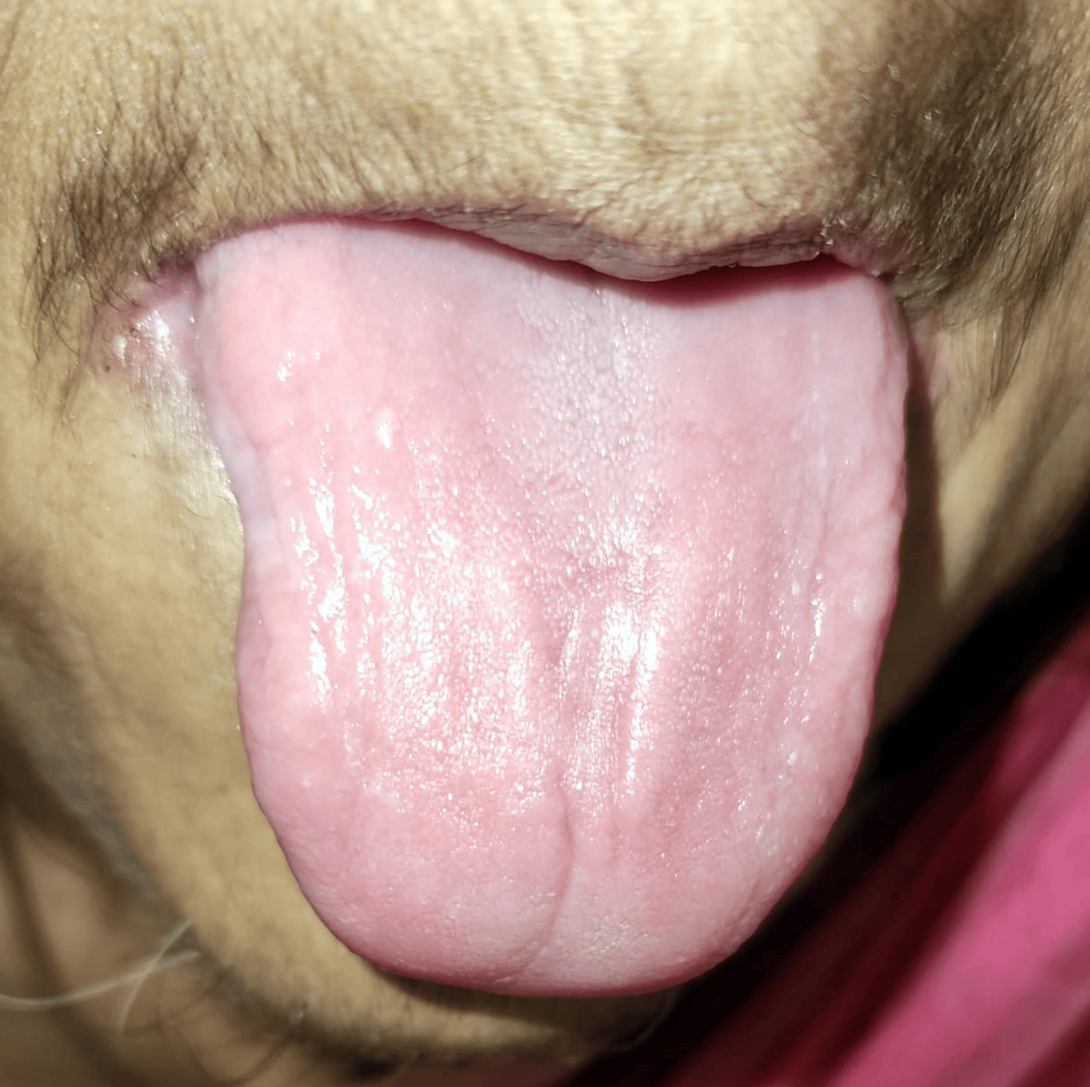
Photograph showing bald tongue. Image credits: Prasanna Sonar

On the tongue's dorsal surface, there was depapillation that suggested a bald tongue as shown in Figure 2. The tongue's texture was smooth and the tongue presented a glossy appearance. 

Pernicious anemia, iron deficiency anemia, and burning mouth syndrome were the differential diagnoses initially made based on the observations. Her blood counts and serum chemical profile were normal. Her serum zinc, iron, and vitamin B12 levels were also within acceptable ranges. Pernicious anemia and iron deficiency anemia were ruled out, which gave the final diagnosis of burning mouth syndrome in association with angular cheilitis. The multifaceted nature of the condition, the absence of a distinct pathophysiological mechanism, and the lack of a defined treatment plan were all covered during patient counseling. The possible connection between persistent anxiety and burning mouth syndrome was also discussed. Through shared decision-making, a referral for cognitive-behavioral therapy with psychologists was made, and the potential benefits of supportive therapy and regular office follow-ups were discussed. Patient education and counseling were done regarding the disease and conservative treatment was advised. The patient was also advised to avoid spicy food as it was an aggravating factor for the symptoms experienced. Also, the patient was encouraged to ensure adequate hydration.

**Figure 3:**
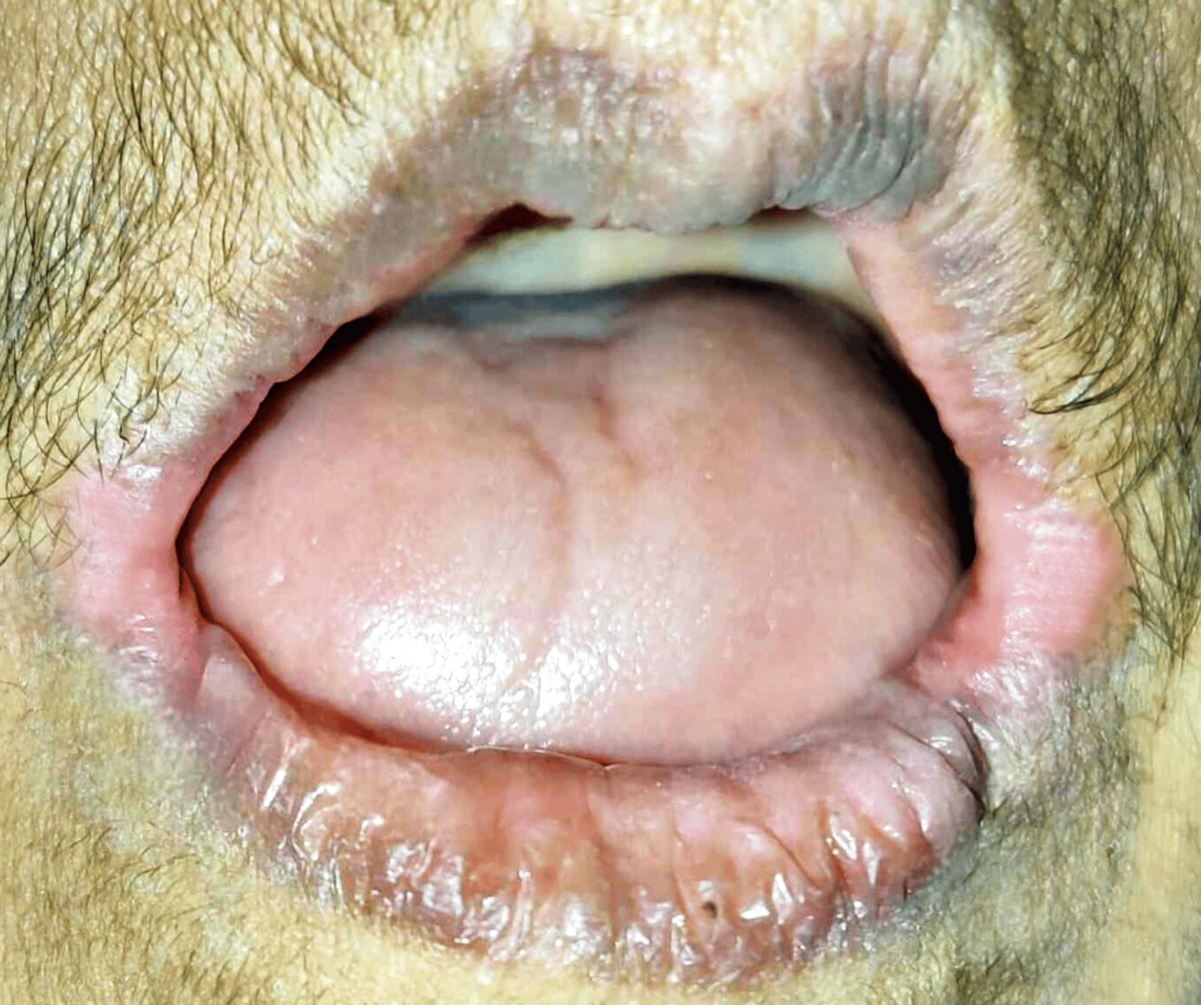
Two-week follow-up visit photograph showing a reduction in erythema on corner of the mouth (bilaterally). Image credits: Prasanna Sonar.

It was recommended to use triphala oral rinse three times a day. It was suggested that the preparation be made fresh each time. The patient was instructed to thoroughly swirl half a spoonful of triphala powder into half a cup of warm water. The administration of clonazepam by swallowing 1 mg pill three times a day for 14 days. For angular cheilitis, topical application of ozonated oil (ADC Inc. Dentozone India, Raigad, Maharashtra, India) was advised three times daily till erythema was completely reduced. The patient was called for follow-up after two weeks, which revealed a reduction in erythema at the corner of the mouth bilaterally as shown in Figure 3. The patient also reported a reduction in burning sensation during her meals. The patient was planned for a complete denture prosthesis and she will be kept on follow-up initially every four to six weeks, contingent upon the severity of symptoms and the response to treatment. Follow-up appointments may be tapered back to once every three to six months once the illness is well-managed.

## Discussion

Burning mouth syndrome (BMS) is defined as an intense discomfort in the tongue or oral mucous membranes, often in the absence of associated clinical and laboratory abnormalities [1]. Up to two-thirds of patients have taste abnormalities, which frequently involve persistent metallic, bitter, or both sensations. Food stimulation frequently lessens the dysgeusic tastes that precede oral burning [3,6,7]. On the other hand, topical anesthesia may cause a decrease in dysgeusic tastes but an increase in oral burning [1]. Although it has been connected to several associated diseases and chronic pain disorders, including headaches and other sites of pain, BMS has not been directly related to any specific medical condition. Additionally, iron deficiency, type 2 diabetes, and a variety of dietary deficits have been linked to BMS [8]. Hormonal changes are believed to be important contributing factors to BMS in postmenopausal women, even if there isn't much conclusive data to support its efficacy in treating the condition [3,9].

Knowing the exact symptoms and the adequate therapies is essential for managing a variety of oral health issues. Menopause frequently results in critical symptoms, which can occasionally have an impact on dental health. If other medical factors are present, hormone replacement treatment may be helpful. A significant contributing factor is inadequate nutrition; deficiencies in zinc, vitamins B1, B2, B6, and other minerals can cause symptoms affecting multiple oral sites, often with mucosal changes [9]. A less painful eating experience is one of the many varied signs of cranial nerve injury, which is often bilateral. Treatment for this condition may involve topical capsaicin application in addition to central pain management techniques like gabapentin, tricyclic antidepressants, or benzodiazepines. Last but not least, adverse drug reactions from prescription medication use may result in timing-related oral symptoms. If possible, changing the prescription regimen can fix these issues. Studies usually support low doses of tricyclic antidepressants, chlordiazepoxide, and clonazepam [10]. Research supports the usefulness of taking gabapentin at a low dosage [11]. The ingestion of a tablet containing 1 mg of clonazepam (an agonist of gamma-aminobutyric acid) can be used in BMS. The use of selective serotonin reuptake inhibitors or other non-serotoninergic antidepressants as treatments has not been shown to have any benefits. Although benzodiazepines can cause oral burning and act as sedative-hypnotics, this appears improbable because clonazepam's maximal effects are frequently observed at lower dosages. Tricyclic antidepressants may also have analgesic effects at low dosages, as evidenced by their ability to lessen chronic pain [12]. Topical capsaicin therapy has proven beneficial for BMS patients as a desensitizing agent [12]. However, capsaicin may not be helpful or tolerated for many patients.

There are potential side effects of using steroid medication as a local application and as a systemic administration. Also, anesthetic gels can be treated only locally and do not affect the underlying cause. This can be well overcome by using triphala, a boon of Ayurveda. "Triphala" is a well-known powdered mixture that has been utilized in Ayurveda since ancient times. Equivalent portions of *Terminalia chebula*, *Emblica officinalis*, and *Terminalia bellirica* make up triphala. Its analgesic, antipyretic, and ulcerogenic properties were found to be similar to that of a nonsteroidal anti-inflammatory drug, indomethacin, in rat experimental models [13]. Supplementing with triphala has been demonstrated to reduce stress. A 48-hour triphala medication can stop the biochemical and behavioral abnormalities brought on by cold stress, including an increase in immobility, as well as an increase in rearing, grooming, and walking activity, as well as a significant spike in corticosterone and lipid oxidation (LPO) levels. However, patient compliance could be a matter of concern regarding triphala oral rinse and ingestion due to its unpleasant taste [14]. Triphala can be made more appealing and patient compliance increased by adding natural sweeteners such as organic honey, stevia or monk fruit extract, coconut sugar, or jaggery, changing the formulation, and teaching patients about the advantages. Assuring that patients adhere to the treatment plan can also be greatly aided by customizing the strategy to meet their requirements and preferences. Chour and Madni, in their clinical study in 2023, have proved that triphala was cheap, easy to adapt, safe, and a better-choice drug in relieving all parameters of aphthous stomatitis [15]. Peterson et al., in a review article, suggested that triphala protected against the negative effects of cold stress, as well as reversed the behavioral and biochemical changes brought on by stress, including elevated corticosterone and lipid peroxidation [16]. In the present case, the patient claimed that all the burning was significantly reduced within 14 days.

A descriptive diagnosis for an inflammatory skin condition with a variety of etiologies that manifests at the labial commissure, or the angle of the mouth, is called angular cheilitis (AC). The oral mucosa and the squamous epithelium of the face meet at the angles of the mouth [4]. Moreover, they serve as a mechanically active hinge for the mouth opening, bearing a greater amount of strain and movement than the surrounding tissue. As a result, the commissures are particularly vulnerable to some stressors. Exposure to chemicals, pathogenic agents, or the environment can result in diffuse cheilitis. AC may be granulomatous cheilitis, exfoliative cheilitis, plasma cell cheilitis, drug-induced cheilitis, infective cheilitis, and nutritional cheilitis [17]. Angular stomatitis, commissural stomatitis, rhagades, and perleche are the other names for AC. Rhagade is a broad word used to describe skin fissuring in motion-prone locations, particularly the nose and labial commissures [5]. Changes in the anatomy of the mouth can have a major impact on lip approximation, which can increase salivary pooling and cause maceration at the labial commissures [18]. Furthermore, an overclosure of the mouth, where the upper lip overlies the lower lip excessively can result from severe tooth wear, edentulous states, or poorly fitting dentures, all of which reduce the vertical dimension of the face and exacerbate salivary pooling. Increased cutaneous wrinkling, such as marionette lines that rely on commissures, might further compromise the structural integrity of the lip. Finally, a special problem for those with Down syndrome is that about 25% of them have AC as a result of macroglossia, which causes the tongue to protrude and increases salivary pooling and drooling. To successfully treat and control the ensuing problems with oral health, it is vital to comprehend these fundamental alterations [5].

Depending on the etiology and clinical circumstances, several therapies are needed for infections at the labial commissures to be effectively managed [6]. Topical fungicidal drugs are often used to treat fungal infections. Options include Gentian violet solution, which can be used twice or three times a day in youngsters, and Nystatin 100,000 units/mL ointment, which can be used twice daily [19]. Other topical antifungals include 2% cream ketoconazole, 1% cream clotrimazole, and 2% cream miconazole, which, because of its gram-positive bacteriostatic action, is frequently used with 1% hydrocortisone for mixed staphylococcal and candidal infections. Topical antiseptics or antibiotics are necessary for bacterial infections, and therapy typically lasts one to two weeks. For an antistaphylococcal regimen, fusidic acid 2% cream can be administered with or without hydrocortisone. Oral antifungals are used in more severe or systemic situations [4,18]. While fluconazole, which is taken orally as 200 mg for one day and then 100 mg every day for up to 14 days, is recommended for moderate to severe instances, particularly in individuals with impaired immune systems, nystatin works well for mild cases. Topical glucocorticoids, such as hydrocortisone 1% ointment and desonide 0.05% ointment, can also be used as an adjunct treatment to antifungal or antibacterial regimens to promote healing and reduce inflammation, or they can be used as monotherapy for inflammatory disorders [20]. In situations of underlying deficiencies, nutritional replacement or supplements may be required to correct vitamin deficiencies, mineral shortages, or general malnutrition. Furthermore, the repair or provision of a full denture to the patient can address the loss of vertical dimension of the face resulting from significant tooth wear, edentulous states, or poorly fitting dentures [21]. When combined, these therapy approaches target the root causes of infection as well as the more general systemic problems that lead to oral health problems.

Ozonated oil, which was used in the present case, offers promising potential for managing oral lesions due to its antimicrobial, anti-inflammatory, and wound-healing properties [22-24]. In 2017, Tarun Kumar et al. carried out a long-term investigation to assess the effectiveness of ozonized olive oil in managing oral lesions and ailments. In patients with aphthous ulcerations, herpes labialis, oral candidiasis, and angular cheilitis, they discovered that all lesions regressed, and in patients with oral lichen planus, the signs and symptoms improved. Not a single subject showed signs of toxicity or adverse effects. The study found that while topical ozone therapy can also have favorable effects without causing toxicity or adverse effects, gaseous ozone therapy is still more effective. As a result, it qualifies as a minimally invasive treatment for immune and infectious conditions of the mouth [25].

The success observed in this case suggests that triphala's anti-inflammatory and antioxidant properties, combined with the antimicrobial and healing effects of ozonated oil, can be an effective treatment strategy for these challenging oral conditions. In the present case, we managed burning sensation in anterior two-thirds of the tongue with triphala. In addition to identifying the best practices for its application, this case study may offer the evidence required to justify its usage and guarantee patient safety. Further clinical studies and trials are recommended to validate these findings and to better understand the mechanisms by which triphala and ozonated oil exert their therapeutic effects. Validating the use of triphala in the treatment of BMS requires clinical trials; these can be carefully designed with a focus on robust outcome measures, allowing researchers to provide the evidence necessary to support or refute these natural therapies and potentially provide new, effective treatment options for patients with BMS. This case underscores the importance of considering integrative and holistic treatment options in managing complex oral conditions, offering a promising avenue for enhancing patient outcomes.

## Conclusions

With an idiopathic origin and no established treatment, burning mouth syndrome is an illness that is challenging to manage and diagnose. This case study illustrates the possible effectiveness of ozonated oil and triphala in treating a patient who presented with both angular cheilitis and burning mouth syndrome. With the complementary approach, which combined modern therapy approaches with traditional Ayurvedic medicine, the patient's overall well-being and symptoms significantly improved.
